# Supplementary material for: A deep learning framework for predicting endometrial cancer from cytopathologic images with different staining styles
Source: PLoS One. 2024 Jul 31;19(7):e0306549. doi: 10.1371/journal.pone.0306549 (PMC11290691; doi:10.1371/journal.pone.0306549)
Supplement: Attachment — Submitted filename: review paper.docx [file pone.0306549.s001.docx]

**Title** : Deep Learning-based Endometrial Cancer Screening System for Multimodal Cytopathology Image

**Summary/Contrubution:** The paper addresses a critical and clinically relevant issue in the field of cancer screening. The development of an automated framework for endometrial cancer detection is commendable, considering the significant impact it can have on early diagnosis and patient outcomes. It is noteworthy that the authors have invested the time and resources to develop the XJTU-EC dataset, which provides a valuable resource for this field.

**Strengths:**

- The authors' dedication to collecting a comprehensive dataset over several years is a significant contribution to the field. The XJTU-EC dataset is expected to facilitate further research in endometrial cancer screening.
- The paper explains the proposed two-stage framework clearly, providing insights into CM-Unet and ECRNet, which are crucial for understanding the methodology. ECRNet uses attention modules and label memory banks to add depth to the methodology. The paper reports that the proposed framework outperforms other classical models.
- The framework has the potential to greatly benefit cytopathologists by enhancing their efficiency. The paper also emphasizes the importance of detection, in cancer and addresses a practical requirement.
- The authors are transparent about the ethical approval process, which adds credibility to the research.

**Areas for improvements:**

- How representative is the XJTU-EC dataset of endometrial cytopathology images, considering it was collected over several years from a single institution? How does it account for potential bias in the dataset? The paper should discuss the generalization potential of the proposed framework to datasets from other medical centers and possibly provide results or insights from external validation on diverse datasets.
- The paper mentions that different medical centers use different staining styles. How robust is the proposed framework to handle extreme variations in staining styles, and can it generalize to datasets from other institutions?
- Could you elaborate on the inter-pathologist agreement in annotating cell clumps? How did you address any disagreements in labeling during the annotation process? What measures were taken to ensure the accuracy and consistency of the segmentation and classification labels given the complexity of cytological images?
- Has there been any validation or real world implementation to evaluate how the proposed framework has affected patient outcomes? Have cytopathologists been engaged in assessing its effectiveness in a clinical setting.

**Further Comments:** In addition to the areas for improvements mentioned above, the authors should also consider the following matters:

- There are some type in the write-up such as in Introduction part “ negative **exsample** pairs”- which should be revised.
- The paper lacks figure numbers, making it challenging to correlate specific references in the text to corresponding figures.
