# Supplementary material for: A deep learning framework for predicting endometrial cancer from cytopathologic images with different staining styles
Source: PLoS One. 2024 Jul 31;19(7):e0306549. doi: 10.1371/journal.pone.0306549 (PMC11290691; doi:10.1371/journal.pone.0306549)
Supplement: Attachment — Submitted filename: Response to Reviewers.docx [file pone.0306549.s002.docx]

Dear Editor and Reviewers,

Thank you very much for your careful review of our manuscript " A Deep Learning Framework for Predicting Endometrial Cancer from Cytopathologic Images with Different Staining Styles" (ID: PONE-D-23-24291). We have revised the manuscript thoroughly according to your comments and suggestions. These main changes in the revised manuscript are highlighted in red. The revisions for grammatical errors and improved sentences are marked in blue. Detailed responses to your comments are provided below.

P.S.: Citations are numbered sequentially in the order in which they appear in the text.

Sincerely,

Dexing Zhong

Academic editor:

1. Please ensure that your manuscript meets PLOS ONE's style requirements, including those for file naming. The PLOS ONE style templates can be found at

https://journals.plos.org/plosone/s/file?id=wjVg/PLOSOne_formatting_sample_main_body.pdf and

https://journals.plos.org/plosone/s/file?id=ba62/PLOSOne_formatting_sample_title_authors_affiliations.pdf.

Responses:

Thank you for your reminder. We have revised the manuscript with reference to the PLOS ONE style template. These main changes in the revised manuscript are highlighted in red. The revisions for grammatical errors and improved sentences are marked in blue. We have determined that the manuscript meets PLOS ONE style requirements, including file naming requirements.

2. During our evaluation of the documents provided, we noted that your ethics approval letter did not cover the entire date range for participant recruitment. Before we can proceed further with the submission, please provide the ethics approval extension documents, along with English translations.

Responses:

Thank you for your suggestion. Our data were provided by the First Affiliated Hospital of Xi'an Jiaotong University. We apologize for the miscommunication with the data provider that led us to mistake the exact time of data collection. We reconfirmed with the data provider and clarified that the data collection lasted for one year, from November 2019 to 2020, which is covered by the ethical approval. After 2020 and up to 2022, two experienced cytopathologists annotated the data. We have revised the relevant parts of the manuscript. Thank you very much for your understanding.

3. Thank you for stating in your Funding Statement:

“This work was supported in part by the National Natural Science Foundation of China

under Grant 62206218, in part by Natural Science Foundation of Zhejiang Province

under Grant LTGG23F030006. Grantees have helped in study design, data collection

and analysis, publication decisions or manuscript preparation.”

Please provide an amended statement that declares *all* the funding or sources of support (whether external or internal to your organization) received during this study, as detailed online in our guide for authors at http://journals.plos.org/plosone/s/submit-now. Please also include the statement “There was no additional external funding received for this study.” in your updated Funding Statement.

Please include your amended Funding Statement within your cover letter. We will change the online submission form on your behalf.

Responses:

Thank you for your suggestion, we have included the Funding Statement at the end of the revised cover letter, which is copied in the attachment below.

***Funding Statement:***

This work was supported in part by the Clinical Research Award of the First Affiliated Hospital of Xi’an Jiaotong University (No. XJTU1AF-CRF-2019-002), in part by the National Natural Science Foundation of China (No. 62206218), in part by the Natural Science Foundation of Zhejiang Province (No. LTGG23F030006). Grantees have helped in study design, data collection and analysis, publication decisions or manuscript preparation.

There was no additional external funding received for this study.

4. We note that you have indicated that data from this study are available upon request. PLOS only allows data to be available upon request if there are legal or ethical restrictions on sharing data publicly. For more information on unacceptable data access restrictions, please see http://journals.plos.org/plosone/s/data-availability#loc-unacceptable-data-access-restrictions.

In your revised cover letter, please address the following prompts:

a) If there are ethical or legal restrictions on sharing a de-identified data set, please explain them in detail (e.g., data contain potentially sensitive information, data are owned by a third-party organization, etc.) and who has imposed them (e.g., an ethics committee). Please also provide contact information for a data access committee, ethics committee, or other institutional body to which data requests may be sent.

b) If there are no restrictions, please upload the minimal anonymized data set necessary to replicate your study findings as either Supporting Information files or to a stable, public repository and provide us with the relevant URLs, DOIs, or accession numbers. For a list of acceptable repositories, please see http://journals.plos.org/plosone/s/data-availability#loc-recommended-repositories.

We will update your Data Availability statement on your behalf to reflect the information you provide.

Responses:

Data cannot be shared publicly because it is patient data and the approvals do not permit us to release the data. Our data are owned by the First Affiliated Hospital of Xi'an Jiaotong University due to ethical committee requirements. If researchers need data, please send a data request email to [xjyfyllh@163.com](mailto:xjyfyllh@163.com), which is the contact information for the Ethics Committee of the First Affiliated Hospital of Xi'an Jiaotong University.

Response to Reviewer #1:

The paper addresses a critical and clinically relevant issue in the field of cancer screening. The development of an automated framework for endometrial cancer detection is commendable, considering the significant impact it can have on early diagnosis and patient outcomes. It is noteworthy that the authors have invested the time and resources to develop the XJTU-EC dataset, which provides a valuable resource for this field.

Responses:

At first, we would like to sincerely thank the anonymous reviewers for your time and concerns to our manuscript, especially your helpful comments and suggestions to improve the quality of our paper. With your kindly help, we have carefully revised the entire manuscript. All the revisions have been clearly highlighted which are visible to the editors and reviewers.

Major comments:

1. How representative is the XJTU-EC dataset of endometrial cytopathology images, considering it was collected over several years from a single institution? How does it account for potential bias in the dataset? The paper should discuss the generalization potential of the proposed framework to datasets from other medical centers and possibly provide results or insights from external validation on diverse datasets.

Response:

We thank the reviewer for the suggestion. In response, we have added a description of the representativeness of our dataset in the Discussion section. Since our dataset contains both papanicolaou and H&E stained images, which are the most common staining modalities for cytology images. Therefore, it can be somewhat considered a representative dataset. In addition, the data were collected across patients of different ages, so the XJTU-EC dataset has more diversity. And the diversity of the training data can help the neural network overcome the effects of potential bias. Our method achieves excellent performance on this dataset, which can demonstrate its generalization ability. We will be collecting more data in the future to further minimize potential bias in the dataset. And we are committed to generalizing the framework presented in this paper to other medical center datasets, and related external validation experiments are in progress. This is part of our future work. The detailed changes (highlighted in red color) can be seen in Discussion in the revised manuscript and the following attachment.

***Discussion (page 19-20, line 404-413):***

Due to the lack of relevant data and the complexity of cell morphology, endometrial cancer screening based on cytopathology is difficult to promote. Therefore, our team collected and annotated WSIs from 139 patients to create the first endometrial cytopathology image dataset, named the XJTU-EC dataset. Since our dataset contains both papanicolaou and H&E stained images, which are the most common staining modalities for cytopathology images. Therefore, it can be somewhat considered a representative dataset. In addition, the data includes patients of different age, so the XJTU-EC dataset has more diversity. Based on this dataset, we investigated the first clinically automated deep learning framework for extracting and identifying normal or cancerous endometrial cell clumps. The results will be presented to cytopathologists as a reference.

***Discussion (page 20-21, line 429-437):***

Finally, there are two limitations of this work. On the one hand, because the data comes from a single institution, our approach is not externally validated on different institutional datasets. Although we have tried to ensure as much diversity as possible in the dataset during the data collection process, and used contrastive learning to enhance the generalization of the screening framework. However, we still lack external validation results from different medical centers. In future work, we will extend our method to other medical center datasets. In addition, annotation has been a challenge due to the scarce number of cytopathologists. We will focus on investigating self-supervised learning to reduce the annotation workload of cytopathologists.

2. The paper mentions that different medical centers use different staining styles. How robust is the proposed framework to handle extreme variations in staining styles, and can it generalize to datasets from other institutions?

Response:

Special thanks to you for your good comments. To the best of our knowledge, papanicolaou staining and H&E staining are the most commonly used staining styles for cytology images. In order to adapt to these two different staining methods, in the cell clump extraction stage, we use the robust Unet as the backbone, which has been previously generalized to many datasets. Based on this, we introduced the CA module and MSS module to further improve the target segmentation accuracy. The segmentation results show that CM-Unet can well ignore the effect of staining style and accurately extract ROIs.

In the cell clump classification stage, we design an ECRNet based on contrastive learning, which considers both instances and label facts. Specifically, different staining style images with the same classification label are considered similar. Thus, it is able to adapt to datasets with changing staining styles. In addition, we learn meaningful and consistent anatomical features through the label contrastive loss *L_con_*, and introduce a label memory bank and a momentum update encoder to maintain encoded feature consistency. Experimental results show that our method achieves excellent performance on mixed staining style datasets, indirectly demonstrating its robustness. We are actively promoting our method to datasets from other institutions, which has been written into our future work. The detailed changes (highlighted in red color) can be seen in Discussion in the revised manuscript and the following attachment.

***Discussion (page 20, line 414-428):***

In order to adapt to different staining styles, in the cell clump extraction stage, we use the robust Unet as the backbone, which has been previously generalized to many datasets. Based on this, we introduced the CA module pay attention to global contextual information, and MSS module to aggregate semantic features at multiple scales. So, our method achieves better segmentation results. Experiments demonstrate that CM-Unet is able to perform well on both H&E-stained images and papanicolaou-stained images.

In the cell clump classification stage, we design an ECRNet based on contrastive learning, which considers both instances and label facts. Specifically, different staining style images with the same classification label are considered similar. Thus, it is able to adapt to datasets with changing staining styles. In addition, we learn meaningful and consistent anatomical features through the label contrastive loss, and introduce a label memory bank and a momentum update encoder to maintain encoded feature consistency. Experimental results show that our method achieves excellent performance on mixed staining style datasets, indirectly demonstrating its robustness. Compared to other methods, ECRNet achieves the best performance in both classification tasks with the two-stage strategy and the one-stage strategy.

3. Could you elaborate on the inter-pathologist agreement in annotating cell clumps? How did you address any disagreements in labeling during the annotation process? What measures were taken to ensure the accuracy and consistency of the segmentation and classification labels given the complexity of cytological images?

Response:

Thank you for your time and patience. We have added a description of the data annotation process in the Methods section. First, a senior cytopathologist annotates the images for segmentation labels, which are reviewed by another cytopathologist. After the review is accurate, we move on to the next process. Undoubtedly, the cell clumps in the negative slides are all negative, but the ones in the positive slides have both negative and positive cell clumps. Therefore, the two cytopathologists again vote on the labeling of each cell clump, when the votes do not agree, they will discuss it. If the discussion fails to result in an accurate diagnosis, the cell clump is discarded. These measures ensure the accuracy and consistency of segmentation and classification labels. The detailed changes (highlighted in red color) can be seen in Methods in the revised manuscript and the following attachment.

***Methods (page 8-9, line 173-192):***

And the image annotation process includes segmentation label annotation and classification label annotation. Segmentation labels were obtained by two experienced pathologists using Adobe Photoshop CC (2019 v20.0.2.30). First, one senior cytopathologist segmented the cell clumps and the results were reviewed by the other cytopathologist. After the review is accurate, the pathologists annotated the cell clumps according to the International Society of Gynecologic Pathologists and the 2014 World Health Organization classification of uterine tumors. All cell clumps were classified into two categories: malignant (atypical cells of undetermined significance, suspected malignant tumor cells, and malignant tumor cells), and benign (non-malignant tumor cells). Benign diagnosis is defined as cell clumps with neat edges, nuclei with oval or spindle shape, and evenly distributed, finely granular chromatin. Malignant diagnosis referred to a three-dimensional appearance, irregular (including dilated, branched, protruding, and papillotubular) edge, with the nucleus poloidal disordering or disappearing (including megakaryocyte appearance, nuclear membrane thickness, and coarse granular or coarse block chromatin). Both benign and malignant tumors were followed up histologically. Undoubtedly, the cell clumps in the negative slides are all negative, but the ones in the positive slides have both negative and positive cell clumps. Therefore, the two cytopathologists again vote on the labeling of each cell clump, when the votes do not agree, they will discuss it. If the discussion fails to result in an accurate diagnosis, the cell clump is discarded. These measures ensure the accuracy and consistency of segmentation and classification labels.

4. Has there been any validation or real world implementation to evaluate how the proposed framework has affected patient outcomes? Have cytopathologists been engaged in assessing its effectiveness in a clinical setting.

Response:

We thank the reviewer for the suggestion. We have two cytopathologists who have been evaluating the effectiveness of the framework in actual clinical applications. After evaluating its accuracy, we tried to apply it in a real setting, and the related article was published [1].

References:

[1] P. Yin, J. An, T. Wang, Y. Wang, Z. Ma, B. Wang, et al. Research on homogenization of AI-assisted medical imaging analysis system for endometrial cells. International Journal of Gynecologic Cancer, 2023. 33(Suppl 4): p. A135-A139.

5. There are some type in the write-up such as in Introduction part “negative exsample pairs”- which should be revised.

Response:

Thank you for your useful comments and suggestions on the language of our manuscript. We have made correction which we hope meet with approval, and modified the manuscript accordingly. We have added more accurate vocabulary and sentence patterns, and removed redundant expressions. These main changes in the revised manuscript are highlighted in red. The revisions for grammatical errors and improved sentences are marked in blue.

6. The paper lacks figure numbers, making it challenging to correlate specific references in the text to corresponding figures.

Response:

Thank you for the reminder. We have uploaded the figures as separate files in Attach Files with reference to PLOS ONE guidelines. Figure files saved as "Fig1.tif", "Fig2.tif ", etc, to make the specific references in the manuscript correspond to the figures. In the manuscript, each figure caption appear directly after the paragraph in which they are first cited.

Reviewer #2:

The authors do deep learning on a set of endometrial lesions based on a variety of available CNN modalities along with their own modality. Their cases were annotated by pathologists and many The data highlights the strengths and weaknesses of currently available algorithms. Their detailed methods could serve as a template to validate other cytology specimens. The code could be validated on larger, institutional and multiinstitutional datasets in the future as it becomes publicly available.

Responses:

At first, we would like to sincerely thank the anonymous reviewer for your time and concerns to our manuscript, especially your helpful comments and suggestions to improve the quality of our paper. We have revised this manuscript and especially paid much attention to your comments and suggestions. All the revisions have been clearly highlighted which are visible to the editors and reviewers.

Major comments:

1. You annotate many different endometrial cancers. Could you provide more information about the tumor types on follow up such as endometrioid, endocervical, mucinous, high grade serous etc? as a pathologist I could have a better understanding of how the cases were annotated and what the algorithm would be evaluating if I knew the different tumor types included similarly for benign is there a histologic follow up.

Responses:

We would like to thank the reviewer again for your carefully review. There are two experienced pathological professors labeled all cytopathological slides and divided them into two classes: malignant (atypical cells of undetermined significance, suspected malignant tumor cells, and malignant tumor cells), and benign (non-malignant tumor cells). A benign diagnosis was defined as cell clumps with neat edges, nuclei with oval or spindle shape, and evenly distributed, finely granular chromatin. Malignant diagnosis referred to a three-dimensional appearance, irregular (including dilated, branched, protruding, and papillotubular) edge, with the nucleus poloidal disordering or disappearing (including megakaryocyte appearance, nuclear membrane thickness, and coarse granular or coarse block chromatin). Only when consistent classification was reached between histology and the two pathologists’ cytology on a sample was the sample considered for the study. Both benign and malignant tumors were followed up histologically. The detailed changes (highlighted in red color) can be seen in Methods in the revised manuscript and the following attachment.

***Methods (page 8-9, line 179-187):***

All cell clumps were classified into two categories: malignant (atypical cells of undetermined significance, suspected malignant tumor cells, and malignant tumor cells), and benign (non-malignant tumor cells). Benign diagnosis is defined as cell clumps with neat edges, nuclei with oval or spindle shape, and evenly distributed, finely granular chromatin. Malignant diagnosis referred to a three-dimensional appearance, irregular (including dilated, branched, protruding, and papillotubular) edge, with the nucleus poloidal disordering or disappearing (including megakaryocyte appearance, nuclear membrane thickness, and coarse granular or coarse block chromatin). Both benign and malignant tumors were followed up histologically.

Reviewer #3:

Major comments:

1. Title: The title is short and not clear.

Responses:

Thank you for your useful comments and suggestions on the language of our manuscript. Regarding the title, according to your recommendations, we changed it to “A Deep Learning Framework for Predicting Endometrial Cancer from Cytopathologic Images with Different Staining Styles”.

2. Abstract:

a) The abstract is too short and it seems as a preface to a report.

b) The objectives of this research are not clear. The authors need to explain exactly what the aim of this research.

Responses:

Thank you for your useful comments and suggestions on the language of our manuscript. We have improved the abstract section and explained the purpose of our study. We aim to develop an endometrial cancer screening algorithm that robustly predicts endometrial cancer on cytopathologic images with different staining styles. This will further advance endometrial cancer screening research and provide early diagnosis for patients. In addition, we improved the linguistic errors and typos present in the abstracts. The detailed changes (highlighted in blue color) can be seen in Abstract in the revised manuscript and the following attachment.

***Abstract:***

Endometrial cancer screening is crucial for clinical treatment. Currently, cytopathologists analyze cytopathology images is considered a popular screening method, but manual diagnosis is time-consuming and laborious. Deep learning can provide objective guidance efficiency. But endometrial cytopathology images often come from different medical centers with different staining styles. It decreases the generalization ability of deep learning models in cytopathology images analysis, leading to poor performance. This study presents a robust automated screening framework for endometrial cancer that can be applied to cytopathology images with different staining styles, and provide an objective diagnostic reference for cytopathologists, thus contributing to clinical treatment. We collected and built the XJTU-EC dataset, the first cytopathology dataset that includes different staining styles. And we propose an efficient two-stage framework for adapting different staining style images, and screening endometrial cancer at the cellular level. Specifically, in the first stage, a novel CM-Unet is utilized to segment cell clumps, with a channel attention (CA) module and a multi-level semantic supervision (MSS) module. It can ignore staining variance and focus on extracting semantic information for segmentation. In the second stage, we propose a robust and effective classification algorithm based on contrastive learning, ECRNet. By momentum-based updating and adding labeled memory banks, it can reduce most of the false negative results. On the XJTU-EC dataset, CM-Unet achieves an excellent segmentation performance, and ECRNet obtains an accuracy of 98.50%, a precision of 99.32% and a sensitivity of 97.67% on the test set, which outperforms other competitive classical models. Our method robustly predicts endometrial cancer on cytopathologic images with different staining styles, which will further advance research in endometrial cancer screening and provide early diagnosis for patients. The code will be available on GitHub.

3. Introduction:

a) In the introduction section, Authors should produce distinctive features mentioning their unique interpretation of the research along with comparative analysis of existing techniques to strengthen their work. Authors can focus on referring more recent journal papers to include further study.

b) There were some linguistic errors and typos.

Responses:

Thank you for your useful comments and suggestions on our manuscript. According to reviewer’s suggestion, we have referred to recent relevant papers and analyzed the related work. On this basis, we have summarized our unique research purpose and significance. In addition, we also corrected the linguistic errors and typos. The detailed revisions can be seen in Introduction section of the revised manuscript, which are also copied in the following attachment.

***Introduction (page 3-4, line 62-86):***

Secondly, diagnosing cytopathological slides is a time-consuming and complex task [16]. Subjective discrepancies and heavy workloads affect the productivity of cytopathologists [17]. As a powerful tool, deep learning can provide objective references for doctors and further improve their work efficiency [18, 19]. Therefore, it is widely used in thyroid cancer [20], cervical cancer [21], liver cancer [22, 23], and other diseases to improve the diagnostic efficiency [24]. And in endometrial diagnosis, deep learning is usually used for segmentation and classification tasks. In the field of segmentation, Erlend Hodneland et al. used a UNet-based 3D convolutional neural network (CNN) to segment endometrial tumors on radiology images, which aimed at generating tumor models for better individualized treatment strategies [25]. Zhiyong Xia et al. designed a dense pyramidal attention U-Net for hysteroscopic images and ultrasound images, which can help doctors to accurately localize the lesion site [26]. In addition, segmentation algorithms are often used to assist in confirming the depth of myometrial infiltration in endometrial cancer [27-30].In the field of classification, Christina Fell et al. used CNNs to classify endometrial histopathology images at the WSI level, which are categorized as “malignant”, “other or benign” and “insufficient” [31]. Sarah Fremond et al. proposed an interpretable endometrial cancer classification system, which can further predict four molecular subtypes of endometrial cancer through self-supervised learning [32]. Min Feng et al. develop a deep learning model for predicting lymph node metastasis from histopathologic images of endometrial cancer, which is believed to predict metastatic status and improve accuracy [33]. In summary, In summary, we note that deep learning models are commonly used for radiology images [34, 35] and histopathology images [36-38] in endometrial studies. Therefore, there is still a lack of endometrial cancer screening algorithms based on cytopathologic images. The aim of our study is to develop a new algorithm that learns cytopathological features and provides an assisted diagnostic strategy.

4. References:

a) The number of references seems sufficient, but most of the references are not recent.

b) References are not well distributed.

Responses:

We would like to thank the reviewer again for your carefully review. We totally agree with this suggestion. In the revised manuscript, we have added some recent relevant work in the references, involving both classic and recent papers. In our future work, we will continue to focus on the research progress in this field. Details (highlighted in red) can be found in the revised manuscript and in the following attachment. The added references are as follows:

***References (page 23-24, line 532-579:***

25.	Hodneland E, Dybvik JA, Wagner-Larsen KS, Šoltészová V, Munthe-Kaas AZ, Fasmer KE, et al. Automated segmentation of endometrial cancer on MR images using deep learning. Scientific Reports. 2021;11(1):179. doi: 10.1038/s41598-020-80068-9.

26.	Xia Z, Zhang L, Liu S, Ran W, Liu Y, Tu J. Deep learning-based hysteroscopic intelligent examination and ultrasound examination for diagnosis of endometrial carcinoma. The Journal of Supercomputing. 2022;78(9):11229-44. doi: 10.1007/s11227-021-04046-2.

27.	Xiong L, Chen C, Lin Y, Mao W, Song Z. A computer-aided determining method for the myometrial infiltration depth of early endometrial cancer on MRI images. BioMedical Engineering OnLine. 2023;22(1):103.

28.	Otani S, Himoto Y, Nishio M, Fujimoto K, Moribata Y, Yakami M, et al. Radiomic machine learning for pretreatment assessment of prognostic risk factors for endometrial cancer and its effects on radiologists' decisions of deep myometrial invasion. Magnetic Resonance Imaging. 2022;85:161-7. doi: https://doi.org/10.1016/j.mri.2021.10.024.

29.	Mao W, Chen C, Gao H, Xiong L, Lin Y. Quantitative evaluation of myometrial infiltration depth ratio for early endometrial cancer based on deep learning. Biomedical Signal Processing and Control. 2023;84:104685. doi: https://doi.org/10.1016/j.bspc.2023.104685.

30.	Dong H-C, Dong H-K, Yu M-H, Lin Y-H, Chang C-C. Using Deep Learning with Convolutional Neural Network Approach to Identify the Invasion Depth of Endometrial Cancer in Myometrium Using MR Images: A Pilot Study. International Journal of Environmental Research and Public Health. 2020;17(16):5993. PubMed PMID: doi:10.3390/ijerph17165993.

31.	Fell C, Mohammadi M, Morrison D, Arandjelović O, Syed S, Konanahalli P, et al. Detection of malignancy in whole slide images of endometrial cancer biopsies using artificial intelligence. Plos one. 2023;18(3):e0282577.

32.	Fremond S, Andani S, Wolf JB, Dijkstra J, Melsbach S, Jobsen JJ, et al. Interpretable deep learning model to predict the molecular classification of endometrial cancer from haematoxylin and eosin-stained whole-slide images: a combined analysis of the PORTEC randomised trials and clinical cohorts. The Lancet Digital Health. 2023;5(2):e71-e82.

33.	Feng M, Zhao Y, Chen J, Zhao T, Mei J, Fan Y, et al. A deep learning model for lymph node metastasis prediction based on digital histopathological images of primary endometrial cancer. Quant Imaging Med Surg. 2023;13(3):1899-913. Epub 2023/03/15. doi: 10.21037/qims-22-220. PubMed PMID: 36915334; PubMed Central PMCID: PMCPMC10006118.

34.	Kurata Y, Nishio M, Moribata Y, Kido A, Himoto Y, Otani S, et al. Automatic segmentation of uterine endometrial cancer on multi-sequence MRI using a convolutional neural network. Scientific Reports. 2021;11(1):14440. doi: 10.1038/s41598-021-93792-7.

35.	Urushibara A, Saida T, Mori K, Ishiguro T, Inoue K, Masumoto T, et al. The efficacy of deep learning models in the diagnosis of endometrial cancer using MRI: a comparison with radiologists. BMC Medical Imaging. 2022;22(1):80. doi: 10.1186/s12880-022-00808-3.

36.	Zhang Y, Chen S, Wang Y, Li J, Xu K, Chen J, et al. Deep learning-based methods for classification of microsatellite instability in endometrial cancer from HE-stained pathological images. Journal of Cancer Research and Clinical Oncology. 2023;149(11):8877-88. doi: 10.1007/s00432-023-04838-4.

37.	Zhang X, Ba W, Zhao X, Wang C, Li Q, Zhang Y, et al. Clinical-grade endometrial cancer detection system via whole-slide images using deep learning. Frontiers in Oncology. 2022;12. doi: 10.3389/fonc.2022.1040238.

38.	Hong R, Liu W, DeLair D, Razavian N, Fenyö D. Predicting endometrial cancer subtypes and molecular features from histopathology images using multi-resolution deep learning models. Cell Reports Medicine. 2021;2(9):100400. doi: https://doi.org/10.1016/j.xcrm.2021.100400.
