# Supplementary material for: A deep learning framework for predicting endometrial cancer from cytopathologic images with different staining styles
Source: PLoS One. 2024 Jul 31;19(7):e0306549. doi: 10.1371/journal.pone.0306549 (PMC11290691; doi:10.1371/journal.pone.0306549)
Supplement: Attachment — Submitted filename: Response to Reviewers.docx [file pone.0306549.s003.docx]

Dear Editor and Reviewers,

Thank you very much for your careful review of our manuscript " A Deep Learning Framework for Predicting Endometrial Cancer from Cytopathologic Images with Different Staining Styles" (ID: PONE-D-23-24291). We have revised the manuscript thoroughly according to your comments and suggestions. These main changes in the revised manuscript are highlighted in red. The revisions for grammatical errors and improved sentences are marked in blue. Detailed responses to your comments are provided below.

P.S.: Citations are numbered sequentially in the order in which they appear in the text.

Sincerely,

Dexing Zhong

Response to Reviewer #1:

Thank you for providing your responses. I have reviewed the answers, and they satisfy my inquiries. I don't have any further comments at this time.

Responses:

We sincerely appreciate all your previous helpful comments and suggestions for improving the quality of our manuscripts. It is also a great honor to receive your encouraging suggestions about our research work, best wishes.

Response to Reviewer #4:

The paper lacks sufficient straightforward considerations to be accepted, as highlighted by Major comment 1 of Reviewer 1 and the corresponding response.

Responses:

At first, we would like to sincerely thank the anonymous reviewers for your time and concerns to our manuscript, especially your helpful comments and suggestions to improve the quality of our paper. With your kindly help, we have carefully revised the entire manuscript. All the revisions have been clearly highlighted which are visible to the editors and reviewers.

Major comments:

1. Reviewer 1 and I share concerns about the potential bias in the XJTU-EC dataset. It is challenging to accept AI research that lacks transparent and adequate teacher data. The authors have stated that additional external validation experiments are being conducted, and until then, this paper will not reach the level of acceptance. The authors consider this as future work, but it is the most crucial task that needs to be done now. Without validation in an external cohort, only a limited number of articles on AI-assisted cytology can be accepted. In 2024, it is not feasible to claim that an AI has been developed solely based on data from a single institution.

Response:

We appreciate your suggestion. Articles focusing on innovations in medicine do require multi-centre validation, however, our work focuses on innovations in algorithms, mainly by proposing a deep learning-based framework for screening endometrial cancer and providing an initial validation of its effectiveness.

On the algorithmic side, due to the difficulty of data collection, there are also some articles that collect data from single institution and this is feasible in terms of algorithmic validation. For example, Sun et al. collected endometrial histopathological images from the Third Affiliated Hospital of Zhengzhou University (a total of 3,306 images) and validated the endometrial four-classification model on the dataset from this institution [1]. Whereas, our endometrial cytopathological images included 3,620 positive images and 2,380 negative images. In addition, Zhang et al. proposed a deep learning model for endometrial lesion classification, which was shown to achieve good results on 1,851 images from Shengjing Hospital of China Medical University [2]. Li et al. collected endometrial cytopathology images at the First Affiliated Hospital of Xi'an Jiaotong University and preliminarily verified the validity of the automated endometrial cancer screening system [3]. Nakayama et al. collected ultrasound images from 20 subjects at Niigata University and used them to train a deep learning-based model suitable for deep vein thrombosis diagnosis [4]. In this study, the main conclusion we draw from the data is that even in the same hospital, the data may be subject to internal biases such as staining differences. In the face of these challenges, our model can effectively overcome internal biases and screen for endometrial cancer cell clumps. We agree that multi-centre validation is a better indication of the effectiveness of our model, and we are actively pursuing relevant data collection and ethical approval, which will be achieved in future work. As for the main goal of this manuscript, which is still to demonstrate the effectiveness of the algorithm in overcoming the internal bias of the data, we initially demonstrated that our algorithm is feasible.

References:

[1] Sun H, Zeng X, Xu T. et al. Computer-Aided Diagnosis in Histopathological Images of the Endometrium Using a Convolutional Neural Network and Attention Mechanisms, IEEE Journal of Biomedical and Health Informatics, vol. 24, no. 6, pp. 1664-1676, June 2020, doi: 10.1109/JBHI.2019.2944977.

[2] Zhang Y, Wang Z, Zhang J. et al. Deep learning model for classifying endometrial lesions. J Transl Med 19, 10 (2021). <https://doi.org/10.1186/s12967-020-02660-x>

[3] Li Q, Wang R, Xie Z, Zhao L, Wang Y, Sun C, Han L, Liu Y, Hou H, Liu C, et al. Clinically Applicable Pathological Diagnosis System for Cell Clumps in Endometrial Cancer Screening via Deep Convolutional Neural Networks. Cancers. 2022; 14(17):4109. https://doi.org/10.3390/cancers14174109

[4] Nakayama Y, Sato M, Okamoto M, Kondo Y, Tamura M, et al. (2023) Deep learning-based classification of adequate sonographic images for self-diagnosing deep vein thrombosis. PLOS ONE 18(3): e0282747. <https://doi.org/10.1371/journal.pone.0282747>

2. It appears that even after the revision, the description of the XJTU dataset is still inadequate. I understand that there are too few cases to start with. I am unsure about what was defined as &quot;the first large cytopathology image dataset&quot;. For reference, the dataset used by Kanavati et al. to develop AI for cervical cytology only had 3121 cases with Papanicolaou staining [PMID: 35267466]. It is not surprising to see different ages included in the study as collecting 139 cases of the same age would be difficult. It is important to provide specific details about the age dispersion to maintain transparency. It&#39;s concerning that 139 cases lack mention of lesions or histology. Could you please provide information about their pregnancy and childbirth history?　 Whether or not age alone creates bias in data from a single center is not clear; exclusion criteria inconsistencies exist, such as allowing ovarian cancer but excluding cervical cancer.

Response:

Thank you for your comments. In response, we have modified confusing descriptions and rewritten the data collection section in the revised manuscript. Specifically, to the best of our knowledge, the XJTU-EC dataset is the first cytopathological image dataset for endometrial cancer screening. In addition, we apologise for the carelessness in our writing and we have rewritten the data collection section in the revised manuscript. In fact, the exclusion criteria during data collection were as follows: (1) diagnosed with suspected pregnancy or pregnancy; (2) diagnosed with acute inflammation of the reproductive system; (3) patients who had undergone hysterectomy for a previous diagnosis of cervical cancer, cervical intraepithelial neoplasia, or ovarian cancer and so on; (4) diagnosed with dysfunctional clotting diseases; and (5) women who body temperature at 37.5°C or higher twice a day were also excluded. Patients who had previously undergone hysterectomy for cervical cancer, cervical intraepithelial neoplasia, or ovarian cancer were excluded. Endometrial cells could not be collected from these patients because they had already undergone hysterectomy.

In addition, we collected the following information from all participants: age, histopathological diagnosis and reproductive history. We recreated Table 1 and added the childbirth history of the patients. Details of the age distribution are shown in Figure 1, as well as specific information on histopathological diagnoses in Table 2.

The detailed changes (highlighted in red color) can be seen in Data collection in the revised manuscript and the following attachment.

***Introduction:***

Firstly, there are no endometrial cytopathology datasets available due to the difficulty in obtaining data and the high cost of high-quality annotation. To combat the challenge, our team collecting 139 cytopathology whole slide images (WSI) with our own designed endometrial sampling device Li Brush (20152660054, Xi'an Meijiajia Medical Technology Co., Ltd., China). Among them, 39 WSIs are papanicolaou stained, and 100 WSIs are hematoxylin and eosin (H&E) stained. These WSIs are annotated by two cytopathologists, thus building a dataset for cytological screening of endometrial cancer. To the best of our knowledge, this is the first cytopathology image dataset for endometrial cancer screening.

***Data collection:***

Patient exclusion criteria were as follows: (1) diagnosed with suspected pregnancy or pregnancy; (2) diagnosed with acute inflammation of the reproductive system; (3) patients who had undergone hysterectomy for a previous diagnosis of cervical cancer, cervical intraepithelial neoplasia, or ovarian cancer and so on; (4) diagnosed with dysfunctional clotting diseases; and (5) women who body temperature at 37.5°C or higher twice a day were also excluded.

The study is approved by the Ethics Committee of the First Affiliated Hospital of Xi'an Jiaotong University (XJTU1AFCRC2019SJ-002), and written consent was obtained from all patients. Minors were not included in the study. And the authors will not have access to information that could identify individual participants. After three years of collection, 139 patients are eventually included in the study and their details are shown in Table 1, which includes the age of the patients, childbirth history, menstrual status, and any other diseases. The detailed information of age distribution is shown in Figure 1. In addition, histopathological diagnosis was also collected and the specific information is shown in Table 2. It is worth mentioning that the protocols used in this study were all in accordance with the ethical principles of the Declaration of Helsinki on Medical Research [43].

**Table 1. Patient Characteristics**

| Characteristics | Number |
| --- | --- |
| **SOURCE** |  |
| IPD | 81 |
| OP | 58 |
| **AGE** |  |
| <40 years old | 32 |
| ≥40 years old | 107 |
| **MENSTRUAL STATUS** |  |
| Premenopausal | 77 |
| Postmenopausal | 35 |
| AUB | 27 |
| **OTHER DISEASE** |  |
| Ovarian cancer | 18 |
| Hypertension | 24 |
| Diabetes | 24 |
| Hormone replacement therapy | 32 |
| **CHILDBIRTH EXPERIENCE** |  |
| Yes | 101 |
| No | 28 |

IPD, Inpatient Department. OP, Outpatient. AUB, Abnormal uterus bleeding. Some information of the patients is missing.

**Fig. 1. The age distribution of the patients.**

Fig. 1. Age distribution figure.

**Table 2. Pathological diagnosis**

| Histological diagnostic results | Number |
| --- | --- |
| Proliferative endometrium | 14 |
| Secretory endometrium | 8 |
| Atrophic endometrium | 10 |
| Mixed endometrium | 2 |
| Endometrial hyperplasia without atypia | 39 |
| Endometrial atypical hyperplasia | 4 |
| **ENDOMETRIAL CARCINOMA** | 62 |
| Endometrioid carcinoma, G1/G2 | 47 |
| Endometrioid carcinoma, G3 | 11 |
| Serous carcinoma | 2 |
| Clear cell carcinoma | 2 |

G1, G2, G3 represent grade 1, grade 2, grade 3 respectively.

Reviewer #5:

Major comments:

1. The channel attention module is similar with the scale-aware distilled decoder proposed in &#39;&#39;COINet: Adaptive Segmentation with Co-Interactive Network for Autonomous Driving&#39;&#39;. This paper should be cited.

Liu, Jie, et al. &quot;COINet: Adaptive Segmentation with Co-Interactive Network for Autonomous Driving.&quot; 2021 IEEE/RSJ International Conference on Intelligent Robots and Systems (IROS). IEEE, 2021.

Responses:

Thank you for your detailed suggestions. We have cited this reference in the Endometrial Cancer Screening section of the revised manuscript. Details (highlighted in red) can be found in the revised manuscript and in the following attachment. The added references are as follows:

***Methods:***

To better accommodate different image staining styles, inspired by [47], we apply CA module at the bottleneck of the encoder-decoder network. The CA module integrates the semantic relationships between different channel mappings, and emphasises strongly interdependent channel mappings by adjusting the weights [48]. As shown in Fig 4.

***References:***

47.	Liu J, Guo X, Li B, Yuan Y, editors. COINet: Adaptive Segmentation with Co-Interactive Network for Autonomous Driving. 2021 IEEE/RSJ International Conference on Intelligent Robots and Systems (IROS); 2021 27 Sept.-1 Oct. 2021.

Reviewer #6:

The paper a framework for endometrial cancer using deep learning, addressing the challenge of analyzing cytopathology images with different staining styles. The framework includes a novel CM-UNet for cell clump segmentation and an ECRNet classification algorithm based on contrastive learning.

Responses:

At first, we would like to sincerely thank the anonymous reviewers for your time and concerns to our manuscript, especially your helpful comments and suggestions to improve the quality of our paper. With your kindly help, we have carefully revised the entire manuscript. All the revisions have been clearly highlighted which are visible to the editors and reviewers.

Major comments:

1. The contribution of proposed work is missing. The authors need to highlight the research gaps and should explicitly mention how proposed framework filled those gaps.

Response:

Thank you for your suggestion. In response, we have emphasized the current research gaps in automated endometrial cancer screening systems in the introductory section and listed the contributions of our work.

First, in recent years, deep learning is widely used in thyroid cancer, cervical cancer, liver cancer, and other diseases to improve the diagnostic efficiency. However, there are very few studies on endometrial cancer screening, and there is a lack of available datasets. Therefore, our team builds a dataset for endometrial cancer cytology screening and implements deep learning to classify endometrial cancer cytology images.

Then, compared with histopathological image segmentation, endometrial cytology images have more noise and sparser semantic features, which pose challenges to the segmentation algorithms. We propose a segmentation model based on the UNet architecture, and in order to better extract the semantic features into the cytology images, the channel attention (CA) module and the multilevel semantic supervision (MSS) module are introduced to obtain more local and global contextual representations.

Finally, considering that images with the same classification label may be represented differently from each other, e.g., variations in staining styles, which may affect the classification model performance. In order to fully utilize the image content information and the labeling information, we propose ECRNet, and introduce the label memory bank to make ECRNet focus more on the class-level discriminative information. Details (highlighted in red color) can be seen in Introduction in the revised manuscript and the following attachment.

***Introduction:***

The main contributions of our study are as follows:

1. Computer-aided diagnostic studies for endometrial cancer screening are scarce and there is a lack of available datasets. Therefore, our team created an endometrial cancer cytology dataset, which was annotated by two cytopathologists. This dataset contains segmentation labels and classification labels that can be used for deep learning.

2. Compared to histopathology image segmentation, endometrial cytology images have more noise and sparser semantic features, which pose a challenge to segmentation algorithms. We propose a segmentation model based on the UNet architecture, and for better extraction of semantic features in cytology images, we introduce the CA module and the MSS module to learn more local and global contextual representations.

3. Considering that images with the same classification label may be represented differently from each other, e.g., variations in staining styles, which may affect the classification model performance. In order to make full use of the image content information and label information, we propose ECRNet and introduce the label memory bank to make ECRNet focus more on the class-level discriminative information.

4. The framework performs efficiently on H&E-stained and papanicolaou-stained cytopathology images, and shows cytopathologists the negative and positive cell clumps. On the test set, it achieved an average accuracy of 98.50%, an area under the curve (AUC) of 93.66% compared to other classical models. The results show that our model can contribute to medical decision-making.

2. In cytopathological images, cells can exhibit diverse shapes and sizes, making it difficult for an algorithm to generalize well across different cell types and imaging conditions. How the proposed method addresses this problem?

Response:

Special thanks to you for your good comments. Your valuable comments are especially appreciated. To the best of our knowledge, in clinical diagnosis, cell clumps are regions of interest (ROI) for cytopathologists, while noise in the background interferes with the diagnostic process of cytopathologists. Therefore, we first apply a segmentation model that excludes the influence of meaningless noise, enabling the model to focus on the cell clumps.

Then, it can be seen that the ROIs vary in shape and size, and the different representations between these ROIs have the potential to affect the performance of the classification model. Therefore, in order to obtain better classification results, the model needs to focus more on class-level discriminative information. We propose the contrastive learning based algorithm ECRNet to classify ROIs. In contrast to current contrastive learning methods that treat different augmentations of the same image as positive pairs, we introduce the label memory bank to preserve the representation information of the image and the corresponding labels. ECRNet treats two instances with the same label as a positive sample pair, and two instances with different labels as a negative sample pair. Thereby, different images with the same semantics are better aggregated in the representation space, while negative sample pairs are separated in the representation space. This makes better use of class-level discriminative information and enhances the generality of the algorithm to some extent.

The detailed changes (highlighted in red color) can be seen in Introduction in the revised manuscript and the following attachment.

***Introduction:***

In clinical diagnosis, cell clumps are regions of interest (ROIs) for cytopathologists, while the background contains unnecessary noises [42]. So, in the first stage, we propose an improved segmentation network CM-UNet, which extracts ROIs from cytopathology images. We introduce a channel attention (CA) module and a multilevel semantic supervision (MSS) module to obtain more local and global contextual representations. In addition, we added novel skip connections to efficiently extract multi-scale features.

In the second stage, we need to classify ROIs to screen positive cell clumps. Since the obtained ROIs vary in shape and size, the different representations among these ROIs may affect the performance of the classification model. We propose the contrastive learning based algorithm ECRNet to classify ROIs. In contrast to current contrastive learning methods that treat different augmentations of the same image as positive pairs, we introduce the label memory bank to preserve the representation information of the image and the corresponding labels. ECRNet treats two instances with the same label as a positive sample pair, and two instances with different labels as a negative sample pair. Thereby, different images with the same semantics are better aggregated in the representation space, while negative sample pairs are separated in the representation space. This makes better use of class-level discriminative information and enhances the generality of the algorithm to some extent.

3. Please include one figure that illustrates the overall flow of the framework. From input image to segmentation output.

Responses:

Thank you for your helpful comments and suggestions on our manuscript. In response, we have redrawn the overall flow of the framework, the details are shown in Fig. 3. It includes detailed information from input images to ROIs. The detailed changes can be seen in the revised manuscript and the following attachment.

***Methods:***

**Fig. 3.** **Pipeline of the proposed framework for cell clumps segmentation.** CM-UNet allows more flexible feature fusion at decoder nodes through densely connected skip connections. L is the loss function. The bold links represent the necessary depth supervision and the light coloured links represent the optional ones.

Fig. 3. Pipeline of the proposed framework for cell clumps segmentation.

4. Please include high resolution images.

Responses:

Thank you for your useful comments and suggestions on our manuscript. As your suggestion, we have replaced all the images with high resolution images and uploaded them in tiff format.

5. Since the framework is solving the segmentation problem, therefore, following references need to discussed in the revised manuscript.

a. An Encoder&ndash;Decoder Deep Learning Framework for Building Footprints Extraction from Aerial Imagery. Arabian Journal for Science and Engineering, pp.1-12.

Responses:

Thank you for your suggestions. We have cited this reference in the revised manuscript. Details (highlighted in red) can be found in the revised manuscript and in the following attachment. The added references are as follows:

***Methods:***

The CM-UNet performed ResNet101 as the backbone, and computes the aggregation of all feature maps at each node by applying dense connections [44-46].

***References:***

46.	Khan SD, Alarabi L, Basalamah S. An Encoder–Decoder Deep Learning Framework for Building Footprints Extraction from Aerial Imagery. Arabian Journal for Science and Engineering. 2023;48(2):1273-84. doi: 10.1007/s13369-022-06768-8.

6. How many parameters are used to train the network?

Responses:

We appreciate your suggestion. In response, we have recreated the relevant tables and added the number of parameters used to train the network. Details (highlighted in red) can be found in the revised manuscript and in the following attachment.

***Segmentation results:***

We applied our segmentation algorithm and other classical segmentation algorithms, such as fully convolutional networks (FCN) [56], UNet [44], UNet++ [45], LinkNet [57], DeepLabV3 [58], and DeepLabV3+ [59] on HE-stained images and papanicolaou-stained images, respectively. The experimental results are shown in Table 3. Our model shows great potential in segmenting cell clumps, with the average Dice value exceeding 0.85. In addition, we conducted ablation experiments, as shown in Table 3, to verify the role played by the CA module and the MSS module in the segmentation model.

**Table 3. Comparison Experiments**

| Model | Dice | Training time | Inference time | Params |
| --- | --- | --- | --- | --- |
| FCN | 0.61 | 2.17h | 0.045s | 270M |
| UNet | 0.75 | 2.50h | 0.022s | 33M |
| UNet++ | 0.85 | 1.49h | 0.029s | 30M |
| LinkNet | 0.79 | **1.21h** | 0.030s | **12M** |
| DeepLabV3 | 0.81 | 2.30h | 0.030s | 54M |
| DeepLabV3+ | 0.85 | 1.80h | 0.025s | 41M |
| UNet++  (with CA) | 0.86 | 1.52h | 0.030s | 31M |
| UNet++  (with MSS) | 0.88 | 1.88h | 0.039s | 33M |
| CM-UNet | **0.89** | 1.90h | 0.039s | 33M |

Comparison experiment of our segmentation algorithm with other classical segmentation algorithms. The inference time is calculated by the single image.

The segmentation results are shown in Fig 6. The first column is the cytological image, the second column is the result of FCN, the third column is the result of UNet, the fourth column is the result of UNet++, the fifth column is the result of LinkNet, the sixth column is the result of DeepLabV3, the seventh column is the result of DeepLabV3+, the eighth column is the result of CM-UNet, and the ninth column is the ground truth annotated by the pathologist. The red boxes represent the over-segmented area, and the yellow boxes represent the under-segmented area. As can be seen from this figure, FCN and UNet have more under-segmentation and fail to identify all cell clumps, which is not suitable for cytopathology image segmentation. Whereas LinkNet and DeepLabV3 tend to over-segment, mistaking mucus and single cells for cell clumps, which does not benefit the subsequent classification task and is therefore also not applicable. The segmentation results of UNet++, DeepLabV3+ and CM-UNet basically conform to the gold standard. However, UNet++ performed moderately well on H&E-stained images but poorly on papanicolaou-stained images, occasionally missing cell clumps. DeepLabV3+, on the other hand, made fewer errors on the papanicolaou-stained images but missed cell clumps on the H&E-stained images. Taken together, the segmentation result of CM-UNet is closer to the annotation of pathologists. It is able to segment all cell clumps and extract ROIs. This demonstrates the performance of our segmentation network.

***Classification results:***

As shown in Table 5, we compared ECRNet with five classical deep learning models. There are the MobileNet [60], the ResNet-101 [61], the Inception-V3 [62], the ViT [63], the ResNeXt-101 [64], the EfficientNet [65], the DenseNet-121 [66], and VGG-16 [67]. Note that all network parameters remain the same as previously described, and the initialization weights are the ImageNet pre-trained weights. These results are obtained in the two-stage framework, which is based on the classification of cell clumps.

**Table 5. Comparison with Baseline Methods**

| Model | Accuracy (%) | Precision (%) | Recall (%) | F1-score (%) | Params |
| --- | --- | --- | --- | --- | --- |
| VGG-16 +SVM | 78.83 | 75.62 | 78.15 | 76.86 | 138M |
| ResNet-101 +SVM | 88.55 | 84.30 | 85.71 | 85.00 | 24M |
| Inception-V3 +SVM | 87.60 | 80.77 | 88.24 | 84.34 | 22M |
| MobileNet-V1 | 82.99 | 79.11 | 74.79 | 76.89 | **5M** |
| Inception-V3 | 82.17 | 81.43 | 83.33 | 82.37 | 22M |
| ViT | 65.00 | 62.43 | 95.58 | 75.52 | 343M |
| ResNeXt-101 | 86.50 | 81.16 | **99.12** | 89.24 | 79M |
| EfficientNet-B7 | 88.50 | 88.79 | 91.15 | 89.96 | 66M |
| ResNet-101 | 92.17 | 97.03 | 87.00 | 91.74 | 24M |
| DenseNet-121 | 93.50 | 92.23 | 95.00 | 93.59 | 8M |
| Ours | **98.50** | **99.32** | 97.67 | **99.33** | 138M |

In this part, we found that the ViT model performs poorly, which may be due to the small size of our dataset and overfitting of the ViT model. In addition, the MobileNet-V1 model also performs poorly, which may be due to the fact that lightweight networks are not good at learning complex cytopathological features. In contrast, the Inception-V3, the ResNeXt-101, the EfficientNet-B7, the ResNet-101, the DenseNet-121, and the ECRNet models performed better, with mean values of accuracy, precision, recall, and F1-scores above 80%. Specifically, ResNeXt-101 has the highest recall, but has 12% less classification accuracy than ECRNet. And ResNet-101 has a precision of 97.03%, second only to ECRNet, which indicates that it has a lower false positive rate. However, the recall of ResNet-101 is only 87.00%, which indicates that it has a higher false negative rate. And it is more likely to miss cancer cell clumps. And ECRNet has the best classification performance, outperforming other classification models in terms of accuracy, precision and F1-score.

7. The details of loss function are missing.

Responses:

Thanks again for your suggestion. In response, in the revised manuscript, we have re-described the loss function for the segmentation model and the loss function for the classification model, adding its details. And added them in the figure. Details (highlighted in red) can be found in the revised manuscript and in the following attachment.

***Endometrial Cancer Screening:***

To solve the gradient disappearance/explosion problem, we introduce the MSS module [49]. By setting appropriate weights for different side output layers, a deeper semantic representation is learned. Assuming that the original input image is represented as *R*, and *d* represents the depth of the CM-UNet model, each output layer *S* performs a 1 × 1 convolution operation, followed by the global average pooling to extract global contextual information. And then we assign a weight factor *α* for each layer of the model. So, the side loss can be defined as:

 (4)

where denotes the relevant parameter of the *i^th^* output layer. And,,, is set sequentially to 0.1, 0.3, 0.6, 0.9.

In addition, we introduce a hybrid segmentation loss to address the class imbalance in the segmentation task:

 (5)

where∈*T*, and∈*P* denote the ground truth and predicted label for class *c* and *n^th^* pixel in the batch. *T* represents the ground truth, and *P* represents the prediction probability. *C* represents the number of categories, and *N* represents the number of pixels in one batch.

Ultimately, the overall loss function of CM-UNet is defined as the weighted sum of the hybrid segmentation loss and the side loss. The final loss function is shown as below:

 (6)

where *d* represents the depth of the CM-UNet model.

***Endometrial Cancer Screening:***

ECRNet consists of two parts: contrastive learning and supervised learning. In contrastive learning, we want to import classification labels in the training data to improve the performance of the classification task. Therefore, we introduce the label memory bank [50]. Two instances with the same label are considered as the same pair, while two instances with different labels are considered as different pairs. This process can be considered as a dictionary look-up task. Given an encoding query *q* (with label *y*), we need to look up the corresponding positive key *k* from a dictionary. Assume that a dictionary of *n* encoded labeled keys {(k_1_, *y_1_*), (k_2_, *y_2_*), ..., (k_n_, *y_n_*)}, for the given encoded query (q, *y*), its label contrastive loss *L_con_* can be calculated as:

 (7)

where is an indicator function that takes the value of 1 if *y* (the label of the *q*) and *y_i_* (the label of the *k_i_*) are the same, otherwise it is 0. *sim*(·) is a similarity function, and τ is a temperature parameter.

In order to store the large number of image representations and labels in the label memory bank, we introduce the momentum update method so as to dynamically construct a large and consistent dictionary [51]. This not only reduces the computational overhead, but also allows the learned representations transfer well to the downstream task. The specific update equation is as follows:

 (8)

Here *m*∈is a momentum coefficient, taking values between [0,1). *θ_k_* is the parameter of the encoder *f_k_*, *θ_q_* is the parameter of the encoder *f_q_*.

In supervised learning, we choose the VGG-16 as the classifier [52, 53]. The cross-entropy loss function is our classifier loss functions and defined as follows:

 (9)

where *Q* is the set of the query image representations, *Y* is the label set, is the predicted probability that the query image *q_i_* is predicted to be *j*.is an indicator function that takes the value of 1 if the query image *q_i_* is classified correctly, otherwise it is 0.

Finally, the ECRNet total loss is calculated as follows:

 (10)

where *β* is a hyperparameter to adjust the relative weight between classification loss and contrastive loss. The value of *β* in general is 0.5.

8. Experiment section is very weak. The authors need to provide more quantitative and qualitative evaluation of the framework as well as comparisons with other reference methods.

Responses:

We thank you again for your careful review. We fully agree with the proposal. In the revised manuscript, we have refined the experimental section, specifically, we have compared our framework with other reference methods and added six comparison experiments. We have rewritten the results tables to include additional assessments. Details (highlighted in red) can be found in the revised manuscript and in the following attachment.

***Segmentation results:***

We applied our segmentation algorithm and other classical segmentation algorithms, such as fully convolutional networks (FCN) [56], UNet [44], UNet++ [45], LinkNet [57], DeepLabV3 [58], and DeepLabV3+ [59] on HE-stained images and papanicolaou-stained images, respectively. The experimental results are shown in Table 3. Our model shows great potential in segmenting cell clumps, with the average Dice value exceeding 0.85. In addition, we conducted ablation experiments, as shown in Table 3, to verify the role played by the CA module and the MSS module in the segmentation model.

**Table 3. Comparison Experiments**

| Model | Dice | Training time | Inference time | Params |
| --- | --- | --- | --- | --- |
| FCN | 0.61 | 2.17h | 0.045s | 270M |
| UNet | 0.75 | 2.50h | 0.022s | 33M |
| UNet++ | 0.85 | **1.49h** | 0.029s | **30M** |
| LinkNet | 0.79 | **1.21h** | 0.030s | **12M** |
| DeepLabV3 | 0.81 | **2.30h** | 0.030s | **54M** |
| DeepLabV3+ | 0.85 | **1.80h** | 0.025s | **41M** |
| UNet++  (with CA) | 0.86 | 1.52h | 0.030s | 31M |
| UNet++  (with MSS) | 0.88 | 1.88h | 0.039s | 33M |
| CM-UNet | **0.89** | 1.90h | 0.039s | 33M |

Comparison experiment of our segmentation algorithm with other classical segmentation algorithms. The inference time is calculated by the single image.

The segmentation results are shown in Fig 6. The first column is the cytological image, the second column is the result of FCN, the third column is the result of UNet, the fourth column is the result of UNet++, the fifth column is the result of LinkNet, the sixth column is the result of DeepLabV3, the seventh column is the result of DeepLabV3+, the eighth column is the result of CM-UNet, and the ninth column is the ground truth annotated by the pathologist. The red boxes represent the over-segmented area, and the yellow boxes represent the under-segmented area. As can be seen from this figure, FCN and UNet have more under-segmentation and fail to identify all cell clumps, which is not suitable for cytopathology image segmentation. Whereas LinkNet and DeepLabV3 tend to over-segment, mistaking mucus and single cells for cell clumps, which does not benefit the subsequent classification task and is therefore also not applicable. The segmentation results of UNet++, DeepLabV3+ and CM-UNet basically conform to the gold standard. However, UNet++ performed moderately well on H&E-stained images but poorly on papanicolaou-stained images, occasionally missing cell clumps. DeepLabV3+, on the other hand, made fewer errors on the papanicolaou-stained images but missed cell clumps on the H&E-stained images. Taken together, the segmentation result of CM-UNet is closer to the annotation of pathologists. It is able to segment all cell clumps and extract ROIs. This demonstrates the performance of our segmentation network.

***Classification results:***

As shown in Table 5, we compared ECRNet with five classical deep learning models. There are the MobileNet [60], the ResNet-101 [61], the Inception-V3 [62], the ViT [63], the ResNeXt-101 [64], the EfficientNet [65], the DenseNet-121 [66], and VGG-16 [67]. Note that all network parameters remain the same as previously described, and the initialization weights are the ImageNet pre-trained weights. These results are obtained in the two-stage framework, which is based on the classification of cell clumps.

**Table 5. Comparison with Baseline Methods**

| Model | Accuracy (%) | Precision (%) | Recall (%) | F1-score (%) | Params |
| --- | --- | --- | --- | --- | --- |
| VGG-16 +SVM | 78.83 | 75.62 | 78.15 | 76.86 | 138M |
| ResNet-101 +SVM | 88.55 | 84.30 | 85.71 | 85.00 | 24M |
| Inception-V3 +SVM | 87.60 | 80.77 | 88.24 | 84.34 | 22M |
| MobileNet-V1 | 82.99 | 79.11 | 74.79 | 76.89 | **5M** |
| Inception-V3 | 82.17 | 81.43 | 83.33 | 82.37 | 22M |
| ViT | 65.00 | 62.43 | 95.58 | 75.52 | 343M |
| ResNeXt-101 | 86.50 | 81.16 | **99.12** | 89.24 | 79M |
| EfficientNet-B7 | 88.50 | 88.79 | 91.15 | 89.96 | 66M |
| ResNet-101 | 92.17 | 97.03 | 87.00 | 91.74 | 24M |
| DenseNet-121 | 93.50 | 92.23 | 95.00 | 93.59 | 8M |
| Ours | **98.50** | **99.32** | 97.67 | **99.33** | 138M |

In this part, we found that the ViT model performs poorly, which may be due to the small size of our dataset and overfitting of the ViT model. In addition, the MobileNet-V1 model also performs poorly, which may be due to the fact that lightweight networks are not good at learning complex cytopathological features. In contrast, the Inception-V3, the ResNeXt-101, the EfficientNet-B7, the ResNet-101, the DenseNet-121, and the ECRNet models performed better, with mean values of accuracy, precision, recall, and F1-scores above 80%. Specifically, ResNeXt-101 has the highest recall, but has 12% less classification accuracy than ECRNet. And ResNet-101 has a precision of 97.03%, second only to ECRNet, which indicates that it has a lower false positive rate. However, the recall of ResNet-101 is only 87.00%, which indicates that it has a higher false negative rate. And it is more likely to miss cancer cell clumps. And ECRNet has the best classification performance, outperforming other classification models in terms of accuracy, precision and F1-score.

9. Please discuss failure cases and also provide their justifications.

Responses:

Thank you for your helpful suggestions. In response, we have discussed the failure cases and analysed them in the revised manuscript.

As shown in Figure 8, we randomly listed 8 correctly classified cell clumps and 8 failure cases in the test set. Of these, the 4 false-negative (missed diagnosis) cases consisted of 1 well-differentiated endometrial adenocarcinoma and 3 poorly differentiated endometrial adenocarcinomas. In contrast, 4 false-positive (over diagnosed) cases included 3 normal cell clumps that were classified as cancer. In some of these classification failure samples, it was difficult for the classification model to extract deeper features because of cell stacking and obscure structural features. In addition, another part of the failed cases showed that the number of cells in the image was small, which was easier to be misclassified by the classification model. This suggests that ECRNet's ability to classify small targets needs to be strengthened in future work.

Details (highlighted in red) can be found in the revised manuscript and in the following attachment. The added references are as follows:

***Classification results:***

Finally, we also discuss the ECRNet classification failure cases. As shown in Figure 8, we randomly listed 8 correctly classified cell clumps and 8 failure cases in the test set. Of these, the 4 false-negative (missed diagnosis) cases consisted of 1 well-differentiated endometrial adenocarcinoma and 3 poorly differentiated endometrial adenocarcinomas. In contrast, 4 false-positive (over diagnosed) cases included 3 normal cell clumps that were classified as cancer. In some of these classification failure samples, it was difficult for the classification model to extract deeper features because of cell stacking and obscure structural features. In addition, another part of the failed cases showed that the number of cells in the image was small, which was easier to be misclassified by the classification model. This suggests that ECRNet's ability to classify small targets needs to be strengthened in future work.

**Fig 8. Examples of correct and incorrect predictions of ECRNet.**

Fig. 8. Presentation of true and false results.

***References:***

**The newly added references are marked in red.**

1.	Urick ME, Bell DW. Clinical actionability of molecular targets in endometrial cancer. Nature Reviews Cancer. 2019;19(9):510-21. doi: 10.1038/s41568-019-0177-x.

2.	Kong A, Johnson N, Kitchener HC, Lawrie TA. Adjuvant radiotherapy for stage I endometrial cancer. Cochrane Database Syst Rev. 2012;2012(4):Cd003916. Epub 2012/04/20. doi: 10.1002/14651858.CD003916.pub4. PubMed PMID: 22513918; PubMed Central PMCID: PMCPMC4164955.

3.	Weiderpass E, Hashim D, Labrèche F. Malignant Tumors of the Female Reproductive System. In: Anttila S, Boffetta P, editors. Occupational Cancers. Cham: Springer International Publishing; 2020. p. 439-53.

4.	Lortet-Tieulent J, Ferlay J, Bray F, Jemal A. International patterns and trends in endometrial cancer incidence, 1978–2013. JNCI: Journal of the National Cancer Institute. 2018;110(4):354-61.

5.	Raglan O, Kalliala I, Markozannes G, Cividini S, Gunter MJ, Nautiyal J, et al. Risk factors for endometrial cancer: An umbrella review of the literature. International Journal of Cancer. 2019;145(7):1719-30. doi: <https://doi.org/10.1002/ijc.31961>.

6.	Nishida N, Murakami F, Kuroda A, Sakamoto Y, Tasaki K, Tasaki S, et al. Clinical utility of endometrial cell block cytology in postmenopausal women. Acta Cytologica. 2017;61(6):441-6.

7.	Pei Z, Cao S, Lu L, Chen W. Direct Cellularity Estimation on Breast Cancer Histopathology Images Using Transfer Learning. Computational and Mathematical Methods in Medicine. 2019;2019:3041250. doi: 10.1155/2019/3041250.

8.	Bao H-j, Chen X, Liu X, Wu W, Li Q-h, Xian J-y, et al. Box C/D snoRNA SNORD89 influences the occurrence and development of endometrial cancer through 2’-O-methylation modification of Bim. Cell Death Discovery. 2022;8(1):309. doi: 10.1038/s41420-022-01102-5.

9.	Liu Y-S, Wang H-M, Gao Y. Controversy on Positive Peritoneal Cytology of Endometrial Carcinoma. Computational and Mathematical Methods in Medicine. 2022;2022:1906769. doi: 10.1155/2022/1906769.

10.	Kondo E, Tabata T, Koduka Y, Nishiura K, Tanida K, Okugawa T, et al. What is the best method of detecting endometrial cancer in outpatients?-endometrial sampling, suction curettage, endometrial cytology. Cytopathology. 2008;19(1):28-33. doi: <https://doi.org/10.1111/j.1365-2303.2007.00509.x>.

11.	Buccoliero AM, Gheri CF, Castiglione F, Garbini F, Barbetti A, Fambrini M, et al. Liquid-based endometrial cytology: cyto-histological correlation in a population of 917 women. Cytopathology. 2007;18(4):241-9. doi: <https://doi.org/10.1111/j.1365-2303.2007.00463.x>.

12.	Wadhwa N, Jatawa SK, Tiwari A. Non-invasive urine based tests for the detection of bladder cancer. Journal of clinical pathology. 2012;65(11):970-5.

13.	Sawaya GF, Smith-McCune K, Kuppermann M. Cervical cancer screening: more choices in 2019. Jama. 2019;321(20):2018-9.

14.	O’Flynn H, Ryan NAJ, Narine N, Shelton D, Rana D, Crosbie EJ. Diagnostic accuracy of cytology for the detection of endometrial cancer in urine and vaginal samples. Nature Communications. 2021;12(1):952. doi: 10.1038/s41467-021-21257-6.

15.	Wang Q, Wang Q, Zhao L, Han L, Sun C, Ma S, et al. Endometrial Cytology as a Method to Improve the Accuracy of Diagnosis of Endometrial Cancer: Case Report and Meta-Analysis. Frontiers in Oncology. 2019;9. doi: 10.3389/fonc.2019.00256.

16.	Ke J, Shen Y, Lu Y, Deng J, Wright JD, Zhang Y, et al. Quantitative analysis of abnormalities in gynecologic cytopathology with deep learning. Laboratory Investigation. 2021;101(4):513-24. doi: 10.1038/s41374-021-00537-1.

17.	Landau MS, Pantanowitz L. Artificial intelligence in cytopathology: a review of the literature and overview of commercial landscape. Journal of the American Society of Cytopathology. 2019;8(4):230-41. doi: <https://doi.org/10.1016/j.jasc.2019.03.003>.

18.	Esteva A, Kuprel B, Novoa RA, Ko J, Swetter SM, Blau HM, et al. Dermatologist-level classification of skin cancer with deep neural networks. Nature. 2017;542(7639):115-8. doi: 10.1038/nature21056.

19.	Lilli L, Giarnieri E, Scardapane S. A Calibrated Multiexit Neural Network for Detecting Urothelial Cancer Cells. Computational and Mathematical Methods in Medicine. 2021;2021:5569458. doi: 10.1155/2021/5569458.

20.	Dov D, Kovalsky SZ, Assaad S, Cohen J, Range DE, Pendse AA, et al. Weakly supervised instance learning for thyroid malignancy prediction from whole slide cytopathology images. Medical Image Analysis. 2021;67:101814. doi: <https://doi.org/10.1016/j.media.2020.101814>.

21.	Ma J, Yu J, Liu S, Chen L, Li X, Feng J, et al. PathSRGAN: Multi-Supervised Super-Resolution for Cytopathological Images Using Generative Adversarial Network. IEEE Transactions on Medical Imaging. 2020;39(9):2920-30. doi: 10.1109/TMI.2020.2980839.

22.	Wei J, Ji Q, Gao Y, Yang X, Guo D, Gu D, et al. A multi-scale, multi-region and attention mechanism-based deep learning framework for prediction of grading in hepatocellular carcinoma. Medical Physics. n/a(n/a). doi: <https://doi.org/10.1002/mp.16127>.

23.	Sun SW, Xu X, Liu QP, Chen JN, Zhu FP, Liu XS, et al. LiSNet: An artificial intelligence -based tool for liver imaging staging of hepatocellular carcinoma aggressiveness. Medical Physics. 2022;49(11):6903-13. doi: <https://doi.org/10.1002/mp.15972>.

24.	Zhao Q, He Y, Wu Y, Huang D, Wang Y, Sun C, et al. Vocal cord lesions classification based on deep convolutional neural network and transfer learning. Medical Physics. 2022;49(1):432-42. doi: <https://doi.org/10.1002/mp.15371>.

25.	Hodneland E, Dybvik JA, Wagner-Larsen KS, Šoltészová V, Munthe-Kaas AZ, Fasmer KE, et al. Automated segmentation of endometrial cancer on MR images using deep learning. Scientific Reports. 2021;11(1):179. doi: 10.1038/s41598-020-80068-9.

26.	Xia Z, Zhang L, Liu S, Ran W, Liu Y, Tu J. Deep learning-based hysteroscopic intelligent examination and ultrasound examination for diagnosis of endometrial carcinoma. The Journal of Supercomputing. 2022;78(9):11229-44. doi: 10.1007/s11227-021-04046-2.

27.	Xiong L, Chen C, Lin Y, Mao W, Song Z. A computer-aided determining method for the myometrial infiltration depth of early endometrial cancer on MRI images. BioMedical Engineering OnLine. 2023;22(1):103.

28.	Otani S, Himoto Y, Nishio M, Fujimoto K, Moribata Y, Yakami M, et al. Radiomic machine learning for pretreatment assessment of prognostic risk factors for endometrial cancer and its effects on radiologists' decisions of deep myometrial invasion. Magnetic Resonance Imaging. 2022;85:161-7. doi: <https://doi.org/10.1016/j.mri.2021.10.024>.

29.	Mao W, Chen C, Gao H, Xiong L, Lin Y. Quantitative evaluation of myometrial infiltration depth ratio for early endometrial cancer based on deep learning. Biomedical Signal Processing and Control. 2023;84:104685. doi: <https://doi.org/10.1016/j.bspc.2023.104685>.

30.	Dong H-C, Dong H-K, Yu M-H, Lin Y-H, Chang C-C. Using Deep Learning with Convolutional Neural Network Approach to Identify the Invasion Depth of Endometrial Cancer in Myometrium Using MR Images: A Pilot Study. International Journal of Environmental Research and Public Health. 2020;17(16):5993. PubMed PMID: doi:10.3390/ijerph17165993.

31.	Fell C, Mohammadi M, Morrison D, Arandjelović O, Syed S, Konanahalli P, et al. Detection of malignancy in whole slide images of endometrial cancer biopsies using artificial intelligence. Plos one. 2023;18(3):e0282577.

32.	Fremond S, Andani S, Wolf JB, Dijkstra J, Melsbach S, Jobsen JJ, et al. Interpretable deep learning model to predict the molecular classification of endometrial cancer from haematoxylin and eosin-stained whole-slide images: a combined analysis of the PORTEC randomised trials and clinical cohorts. The Lancet Digital Health. 2023;5(2):e71-e82.

33.	Feng M, Zhao Y, Chen J, Zhao T, Mei J, Fan Y, et al. A deep learning model for lymph node metastasis prediction based on digital histopathological images of primary endometrial cancer. Quant Imaging Med Surg. 2023;13(3):1899-913. Epub 2023/03/15. doi: 10.21037/qims-22-220. PubMed PMID: 36915334; PubMed Central PMCID: PMCPMC10006118.

34.	Kurata Y, Nishio M, Moribata Y, Kido A, Himoto Y, Otani S, et al. Automatic segmentation of uterine endometrial cancer on multi-sequence MRI using a convolutional neural network. Scientific Reports. 2021;11(1):14440. doi: 10.1038/s41598-021-93792-7.

35.	Urushibara A, Saida T, Mori K, Ishiguro T, Inoue K, Masumoto T, et al. The efficacy of deep learning models in the diagnosis of endometrial cancer using MRI: a comparison with radiologists. BMC Medical Imaging. 2022;22(1):80. doi: 10.1186/s12880-022-00808-3.

36.	Zhang Y, Chen S, Wang Y, Li J, Xu K, Chen J, et al. Deep learning-based methods for classification of microsatellite instability in endometrial cancer from HE-stained pathological images. Journal of Cancer Research and Clinical Oncology. 2023;149(11):8877-88. doi: 10.1007/s00432-023-04838-4.

37.	Zhang X, Ba W, Zhao X, Wang C, Li Q, Zhang Y, et al. Clinical-grade endometrial cancer detection system via whole-slide images using deep learning. Frontiers in Oncology. 2022;12. doi: 10.3389/fonc.2022.1040238.

38.	Hong R, Liu W, DeLair D, Razavian N, Fenyö D. Predicting endometrial cancer subtypes and molecular features from histopathology images using multi-resolution deep learning models. Cell Reports Medicine. 2021;2(9):100400. doi: <https://doi.org/10.1016/j.xcrm.2021.100400>.

39.	Lin H, Chen H, Wang X, Wang Q, Wang L, Heng P-A. Dual-path network with synergistic grouping loss and evidence driven risk stratification for whole slide cervical image analysis. Medical Image Analysis. 2021;69:101955. doi: <https://doi.org/10.1016/j.media.2021.101955>.

40.	Shaban MT, Baur C, Navab N, Albarqouni S, editors. Staingan: Stain Style Transfer for Digital Histological Images. 2019 IEEE 16th International Symposium on Biomedical Imaging (ISBI 2019); 2019 8-11 April 2019.

41.	Geng X, Liu X, Cheng S, Zeng S. Cervical cytopathology image refocusing via multi-scale attention features and domain normalization. Medical Image Analysis. 2022;81:102566. doi: <https://doi.org/10.1016/j.media.2022.102566>.

42.	Maksem JA, Meiers I, Robboy SJ. A primer of endometrial cytology with histological correlation. Diagnostic cytopathology. 2007;35(12):817-44.

43.	Association WM. World Medical Association Declaration of Helsinki: ethical principles for medical research involving human subjects. Jama. 2013;310(20):2191-4.

44.	Ronneberger O, Fischer P, Brox T, editors. U-net: Convolutional networks for biomedical image segmentation. International Conference on Medical image computing and computer-assisted intervention; 2015: Springer.

45.	Zhou Z, Siddiquee MMR, Tajbakhsh N, Liang J. UNet++: Redesigning Skip Connections to Exploit Multiscale Features in Image Segmentation. IEEE Transactions on Medical Imaging. 2020;39(6):1856-67. doi: 10.1109/TMI.2019.2959609.

46.	Khan SD, Alarabi L, Basalamah S. An Encoder–Decoder Deep Learning Framework for Building Footprints Extraction from Aerial Imagery. Arabian Journal for Science and Engineering. 2023;48(2):1273-84. doi: 10.1007/s13369-022-06768-8.

47.	Liu J, Guo X, Li B, Yuan Y, editors. COINet: Adaptive Segmentation with Co-Interactive Network for Autonomous Driving. 2021 IEEE/RSJ International Conference on Intelligent Robots and Systems (IROS); 2021 27 Sept.-1 Oct. 2021.

48.	Fu J, Liu J, Tian H, Li Y, Bao Y, Fang Z, et al., editors. Dual Attention Network for Scene Segmentation. 2019 IEEE/CVF Conference on Computer Vision and Pattern Recognition (CVPR); 2019 15-20 June 2019.

49.	Wu H, Wang W, Zhong J, Lei B, Wen Z, Qin J. SCS-Net: A Scale and Context Sensitive Network for Retinal Vessel Segmentation. Medical Image Analysis. 2021;70:102025. doi: <https://doi.org/10.1016/j.media.2021.102025>.

50.	Ren Y, Bai J, Zhang J, editors. Label Contrastive Coding Based Graph Neural Network for Graph Classification. Database Systems for Advanced Applications; 2021 2021//; Cham: Springer International Publishing.

51.	He K, Fan H, Wu Y, Xie S, Girshick R, editors. Momentum Contrast for Unsupervised Visual Representation Learning. 2020 IEEE/CVF Conference on Computer Vision and Pattern Recognition (CVPR); 2020 13-19 June 2020.

52.	Woo S, Park J, Lee J-Y, Kweon IS, editors. CBAM: Convolutional Block Attention Module. Computer Vision – ECCV 2018; 2018 2018//; Cham: Springer International Publishing.

53.	Liu S, Deng W, editors. Very deep convolutional neural network based image classification using small training sample size. 2015 3rd IAPR Asian Conference on Pattern Recognition (ACPR); 2015 3-6 Nov. 2015.

54.	Dice LR. Measures of the Amount of Ecologic Association Between Species. Ecology. 1945;26(3):297-302. doi: <https://doi.org/10.2307/1932409>.

55.	Kingma DP, Ba J. Adam: A method for stochastic optimization. arXiv preprint arXiv:14126980. 2014.

56.	Shelhamer E, Long J, Darrell T. Fully Convolutional Networks for Semantic Segmentation. IEEE Transactions on Pattern Analysis and Machine Intelligence. 2017;39(4):640-51. doi: 10.1109/TPAMI.2016.2572683.

57.	Chaurasia A, Culurciello E, editors. LinkNet: Exploiting encoder representations for efficient semantic segmentation. 2017 IEEE Visual Communications and Image Processing (VCIP); 2017 10-13 Dec. 2017.

58.	Chen L-C, Papandreou G, Schroff F, Adam H. Rethinking atrous convolution for semantic image segmentation. arXiv preprint arXiv:170605587. 2017.

59.	Chen L-C, Zhu Y, Papandreou G, Schroff F, Adam H, editors. Encoder-decoder with atrous separable convolution for semantic image segmentation. Proceedings of the European conference on computer vision (ECCV); 2018.

60.	Howard AG, Zhu M, Chen B, Kalenichenko D, Wang W, Weyand T, et al. Mobilenets: Efficient convolutional neural networks for mobile vision applications. arXiv preprint arXiv:170404861. 2017.

61.	He K, Zhang X, Ren S, Sun J, editors. Deep residual learning for image recognition. Proceedings of the IEEE conference on computer vision and pattern recognition; 2016.

62.	Szegedy C, Vanhoucke V, Ioffe S, Shlens J, Wojna Z, editors. Rethinking the inception architecture for computer vision. Proceedings of the IEEE conference on computer vision and pattern recognition; 2016.

63.	Dosovitskiy A, Beyer L, Kolesnikov A, Weissenborn D, Zhai X, Unterthiner T, et al. An image is worth 16x16 words: Transformers for image recognition at scale. arXiv preprint arXiv:201011929. 2020.

64.	Xie S, Girshick R, Dollár P, Tu Z, He K, editors. Aggregated Residual Transformations for Deep Neural Networks. 2017 IEEE Conference on Computer Vision and Pattern Recognition (CVPR); 2017 21-26 July 2017.

65.	Tan M, Le Q, editors. Efficientnet: Rethinking model scaling for convolutional neural networks. International conference on machine learning; 2019: PMLR.

66.	Huang G, Liu Z, Van Der Maaten L, Weinberger KQ, editors. Densely connected convolutional networks. Proceedings of the IEEE conference on computer vision and pattern recognition; 2017.

67.	Simonyan K, Zisserman A. Very deep convolutional networks for large-scale image recognition. arXiv preprint arXiv:14091556. 2014.

68.	Xu Y, Jia Z, Wang LB, Ai Y, Zhang F, Lai M, et al. Large scale tissue histopathology image classification, segmentation, and visualization via deep convolutional activation features. BMC Bioinformatics. 2017;18(1):281. Epub 2017/05/28. doi: 10.1186/s12859-017-1685-x. PubMed PMID: 28549410; PubMed Central PMCID: PMCPMC5446756.
