# Supplementary material for: A deep learning framework for predicting endometrial cancer from cytopathologic images with different staining styles
Source: PLoS One. 2024 Jul 31;19(7):e0306549. doi: 10.1371/journal.pone.0306549 (PMC11290691; doi:10.1371/journal.pone.0306549)
Supplement: Attachment — Submitted filename: Response to Reviewers.docx [file pone.0306549.s004.docx]

Dear Editor and Reviewers,

Thank you very much for your careful review of our manuscript " A Deep Learning Framework for Predicting Endometrial Cancer from Cytopathologic Images with Different Staining Styles" (ID: PONE-D-23-24291). We have revised the manuscript thoroughly according to your comments and suggestions. These main changes in the revised manuscript are highlighted in red. The revisions for grammatical errors and improved sentences are marked in blue. Detailed responses to your comments are provided below.

P.S.: Citations are numbered sequentially in the order in which they appear in the text.

Sincerely,

Dexing Zhong

Response to Reviewer #4:

The author was unable to respond to the comments made by reviewer #4. In 2024, medical machine learning research with a small number of data as teachers and without external validation should not be considered as a research paper, or at the very least, should not be published in PLOS One.

Responses:

Thank you for your suggestion. In response, we conducted an external validation of the paper's algorithm using the public dataset. The externally validated data were obtained from the public data platform, AIstudio, accessible via the Internet. It is worth noting that the data used for external validation came from the public dataset and did not contain segmentation labels, so this external validation mainly evaluated the classification performance of ECRNet.

The dataset used for external validation consisted of 848 negative endometrial cytopathology images and 785 positive endometrial cytopathology images. The ratio of negative to positive images was 1.08:1. All images are papanicolaou stained images. During this external validation, we also chose four classification models (the ResNet-101 model, the DenseNet-121 model, the EfficientNet-B7 model, and the ResNeXt-101 model) to compare with our model. This is because these four models perform well in the classification task, second only to ECRNet. Specifically, these models above were first trained using images (1024 × 1024 pixels) from the XJTU-EC dataset instead of cell clumps (ROIs), and then tested directly on an external dataset. The specific results are shown in Table 6 as following:

Table 6. External validation comparison results

| Model | Accuracy (%) | Precision (%) | Recall (%) | F1-score (%) |
| --- | --- | --- | --- | --- |
| ResNeXt-101 | 64.50 | 86.20 | 44.20 | 58.44 |
| EfficientNet-B7 | 83.53 | 78.72 | 59.2 | 67.58 |
| ResNet-101 | 73.50 | **100.00** | 53.10 | 69.37 |
| DenseNet-121 | 80.10 | 77.20 | 67.20 | 71.90 |
| Ours | **95.32** | 94.57 | **96.17** | **95.37** |

In this external validation experiment, ResNet-101 has the highest precision of 100%, which means it has no false positives in the external validation. However, both ResNeXt-101, EfficientNet-B7 ResNet-101, and DenseNet-121 have low recall, which means they can easily miss screening positive patients. This is unacceptable for clinical tasks. In contrast, our model achieved the highest recall of 96.17%. In addition, ECRNet has the highest accuracy of 95.32%, followed by the DenseNet classifier with 83.53%. It is worth noting that ECRNet significantly outperforms the other four classifiers in terms of the F1 score. The F1 score is the reconciled average of precision and recall, which combines the information of precision and recall to provide a more comprehensive assessment of the performance of the classifiers, and the higher the F1 score, the better the performance of the classifiers. The higher the F1 score, the better the performance of the classifier. In summary, ECRNet has the best performance in this external validation experiment. The detailed changes (highlighted in red color) can be seen in Experiments and Results in the revised manuscript.

Finally, regarding the dataset of this manuscript, we are the first endometrial cytopathology dataset that contains segmentation and classification labels. Since making segmentation and classification labels is a laborious and time-consuming process, expanding the dataset will take more time, and we will definitely refine the information in the future. To the best of our knowledge, Koriakina et al. [1] proposed a deep multi-instance learning-based method for oral cancer detection, with data collected from a total of 24 patients; Guha et al. [2] collected 135 patients for a machine learning study of the liver; and Alhassan [3] proposed a deep learning-based method for breast cancer classification, trained and tested on a publicly available dataset of a total of 82 patients, with no external validation.

Previously, there was a lack of deep learning-based research on endometrial cancer cytopathology images, and our work is an initial exploration in automated endometrial cytopathology screening. In addition, our model can overcome the challenge of staining bias in clinical images and has been validated in external data with some significance.

Once again, we would like to express our gratitude for your understanding and sincerely hope for your approval.

**References:**

[1] Koriakina N, Sladoje N, Bašić V, et al. Deep multiple instance learning versus conventional deep single instance learning for interpretable oral cancer detection[J]. Plos one, 2024, 19(4): e0302169.

[2] Guha S, Ibrahim A, Wu Q, et al. Machine learning-based identification of contrast-enhancement phase of computed tomography scans[J]. Plos one, 2024, 19(2): e0294581.

[3] Alhassan A M. An improved breast cancer classification with hybrid chaotic sand cat and Remora Optimization feature selection algorithm[J]. Plos one, 2024, 19(4): e0300622.
