# Supplementary material for: A deep learning framework for predicting endometrial cancer from cytopathologic images with different staining styles
Source: PLoS One. 2024 Jul 31;19(7):e0306549. doi: 10.1371/journal.pone.0306549 (PMC11290691; doi:10.1371/journal.pone.0306549)
Supplement: Attachment — Submitted filename: Response to Reviewers.docx [file pone.0306549.s005.docx]

Dear Editor and Reviewers,

Thank you very much for your careful review of our manuscript " A Deep Learning Framework for Predicting Endometrial Cancer from Cytopathologic Images with Different Staining Styles" (ID: PONE-D-23-24291). We have revised the manuscript thoroughly according to your comments and suggestions. These main changes in the revised manuscript are highlighted in red. The revisions for grammatical errors and improved sentences are marked in blue. Detailed responses to your comments are provided below.

P.S.: Citations are numbered sequentially in the order in which they appear in the text.

Sincerely,

Dexing Zhong

Response to Reviewer #4:

Thank you for your kind response to my repeated requests for revisions. I believe the results of the external validation are good enough to publish a paper on this AI and inquire about it with society. However, I would like you to add one more reference (reference URL) to the newly added sentence in line 438, which mentions "AIstudio, accessible via the Internet." Please include the URL along with the date of access, in the format of a literature citation. If this is completed, I recommend accepting this paper.

Responses:

Thank you for your kind suggestion. In response, we have added the reference URL [69] to the newly added sentence in line 438, referencing the example format provided by PLOS One. Specific changes (highlighted in red) can be found in the revised manuscript and in the following annexes. Finally, we would like to thank you or providing us with valuable revision opportunities to improve the quality of our paper.

***Experiments and Results:***

In addition, we conducted an external validation of the paper's algorithm using the public dataset. The externally validated data were obtained from the public data platform, AIstudio, accessible via the Internet [69]. It is worth noting that the data used for external validation came from the public dataset and did not contain segmentation labels, so this external validation mainly evaluated the classification performance of ECRNet.

***References:***

69.	Endometrial Cancer; 2024 [cited 2024 Jun 4]. Database: AIstudio [Internet]. Available from: https://aistudio.baidu.com/datasetdetail/273988.
